# Individual patient data meta-analysis for the clinical assessment of coronary computed tomography angiography: protocol of the Collaborative Meta-Analysis of Cardiac CT (CoMe-CCT)

**DOI:** 10.1186/2046-4053-2-13

**Published:** 2013-02-15

**Authors:** Georg M Schuetz, Peter Schlattmann, Stephan Achenbach, Matthew Budoff, Mario J Garcia, Robert Roehle, Gianluca Pontone, Willem Bob Meijboom, Daniele Andreini, Hatem Alkadhi, Lily Honoris, Nuno Bettencourt, Jörg Hausleiter, Sebastian Leschka, Bernhard L Gerber, Matthijs FL Meijs, Abbas Arjmand Shabestari, Akira Sato, Elke Zimmermann, Uwe J Schoepf, Axel Diederichsen, David A Halon, Vladimir Mendoza-Rodriguez, Ashraf Hamdan, Bjarne L Nørgaard, Harald Brodoefel, Kristian A Øvrehus, Shona MM Jenkins, Bjørn A Halvorsen, Johannes Rixe, Mehraj Sheikh, Christoph Langer, Eugenio Martuscelli, Andrea Romagnoli, Arthur JHA Scholte, Roy P Marcus, Geir R Ulimoen, Koen Nieman, Hans Mickley, Konstantin Nikolaou, Jean-Claude Tardif, Thorsten RC Johnson, Simone Muraglia, Benjamin JW Chow, David Maintz, Michael Laule, Marc Dewey

**Affiliations:** 1 Department of Radiology, Charité - Universitätsmedizin Berlin Campus Mitte, Humboldt-Universität zu Berlin, Freie Universität Berlin, Charitéplatz 1, Berlin 10117, Germany; 2 Department of Medical Statistics, Informatics and Documentation (PS), University Hospital of Friedrich Schiller University Jena, Jena, Germany; 3 Medizinische Klinik 2, Universitätsklinikum Erlangen, Ulmenweg 18, Erlangen, 91054, Germany; 4 Los Angeles Biomedical Research Institute, 1124 W Carson Street, Torrance, CA, 90502, USA; 5 Division of Cardiology, Montefiore Medical Center - Albert Einstein College of Medicine, 211 East 210th Street, Bronx, NY, 10467, USA; 6 Centro Cardiologico Monzino, IRCCS, Via C. Parea 4, Milan, 20134, Italy; 7 Department of Cardiology, Erasmus Medical Center, ‘s Gravendijkwal 230, Postbus 2040, Rotterdam, CA, 3000, The Netherlands; 8 Institute of Diagnostic and Interventional Radiology, University Hospital Zurich, Zurich, Switzerland; 9 Cardiovascular CT and Cardiac Magnetic Resonance Laboratory, Cardiovascular Diagnostic and Intervention Unit - Department of Cardiology, Centro Hospitalar de Vila Nova de Gaia Hospita, Rua Conceição Fernandes, V.N. Gaia, 4434-502, Portugal; 10 Klinik für Herz- und Kreislauferkrankungen im Erwachsenenalter, Deutsches Herzzentrum München, Klinik an der Technischen Universität München, München, Germany; 11 Institute of Radiology, Kantonsspital St. Gallen, Rorschacher Strasse 95, St. Gallen, 9007, Switzerland; 12 Department of Cardiology, Cliniques Universitaires St. Luc, Universite Catholique de Louvain, Brussels, Belgium; 13 Department of Cardiology, University Medical Center Utrecht, H 100, 3584 CX,, Utrecht, HP E03.511, The Netherlands; 14 Department of Radiology, Modarres Hospital, Shahid Beheshti University of Medical Sciences, Tehran, Iran; 15 Cardiovascular Division, Faculty of Medicine, University of Tsukuba, 1-1-1 Tennodai, Tsukuba, Ibaraki, 305-8575, Japan; 16 Department of Radiology and Radiological Science, Medical University of South Carolina, Charleston, SC, USA; 17 Department of Cardiology, Odense University Hospital, Sdr. Boulevard 29, Odense C, 5000, Denmark; 18 Department of Cardiovascular Medicine, Lady Davis Carmel Medical Center, Haifa, Israel; 19 National Institute of Cardiology and Cardiovascular Surgery, ”Manuel Fajardo” Medical Sciences Faculty, Medical Sciences University of Havana, Tomography Department, Vedado, Plaza de La Revoluciòn, The Havana, Cuba; 20 Department of Internal Medicine/Cardiology, Deutsches Herzzentrum Berlin, Berlin, Germany; 21 Heart Center, Chaim Sheba Medical Center, Tel Hashomer, Sackler Faculty of Medicine, Tel-Aviv University, Tel Hashomer, Israel; 22 Department of Cardiology B, Aarhus University Hospital Skejby, Aarhus N, DK- 8200, Denmark; 23 University Department of Radiology, University Hospital Tübingen, Hoppe-Seyler-Straße 3, Tübingen, 72076, Germany; 24 Department of Cardiology, Vejle Hospital, Vejle, Denmark; 25 Department of Cardiology, Glasgow Royal Infirmary, Glasgow, UK; 26 Department of Cardiology, Ostfold County Hospital, Fredrikstad, N-1603, Norway; 27 Medizinische Klinik I (Kardiologie, Angiologie), Universitätsklinikum Giessen und Marburg GmbH, Standort Giessen, Klinikstr. 33, Giessen, 35392, Germany; 28 Department of Radiology, Faculty of Medicine, Kuwait University, PO Box 24923, Safat, 13110, Kuwait; 29 Klinik für Innere Medizin III mit Schwerpunkt Kardiologie und Angiologie, UKSH, Campus Kiel, Schittenhelmstr. 12, Kiel, D-24105, Germany; 30 Kardiologische Klinik, Herz- und Diabeteszentrum Nordrhein-Westfalen, Universitätsklinik der Ruhr-Universität Bochum, Georgstr. 11, Bad Oeynhausen, 32545, Germany; 31 Division of Cardiology, Department of Internal Medicine, University of Rome Tor Vergata, Viale Oxford 81, Rome, 00133, Italy; 32 Department of Radiology, University of Rome Tor Vergata, Viale Oxford 81, Rome, 00133, Italy; 33 Department of Cardiology, Leiden University Medical Center, Albinusdreef 2, P.O. Box 9600, 2300, RC Leiden, the Netherlands; 34 Department of Clinical Radiology, Ludwig-Maximilians-University of Munich, Marchioninistrasse 15, Munich, 81377, Germany; 35 Department of Radiology, Akershus University Hospital, Lorenskog, Norway; 36 Department of Radiology, Erasmus University Medical Center, 's Gravendijkwal 230, Rotterdam, CE, 3015, The Netherlands; 37 Montreal Heart Institute, Université de Montréal, 5000 Belanger Street, Montreal, PQ H1T 1C8, Canada; 38 Department of Cardiology, S. Chiara Hospital, L.go Medaglie d’Oro 1, Trento, 38100, Italy; 39 University of Ottawa Heart Institute, 40 Ruskin Street, Ottawa, ON K1Y 4W7, Canada; 40 Department of Radiology, University of Cologne, Kerpener Str. 62, Köln, 50937, Germany; 41 Department of Radiology, University of Münster, Albert-Schweitzer-Campus 1, Münster, 48149, Germany; 42 Department of Cardiology, Charité – Universitätsmedizin Berlin Campus Mitte, Humboldt-Universität zu Berlin, Freie Universität Berlin, Charitéplatz 1, Berlin, 10117, Germany

**Keywords:** Collaborative meta-analysis on cardiac CT, CoMe-CCT, Coronary CT angiography, Individual patient data meta-analysis, IPD, Positive and negative predictive value, Pretest likelihood, Sensitivity and specificity, Study protocol

## Abstract

**Background:**

Coronary computed tomography angiography has become the foremost noninvasive imaging modality of the coronary arteries and is used as an alternative to the reference standard, conventional coronary angiography, for direct visualization and detection of coronary artery stenoses in patients with suspected coronary artery disease. Nevertheless, there is considerable debate regarding the optimal target population to maximize clinical performance and patient benefit. The most obvious indication for noninvasive coronary computed tomography angiography in patients with suspected coronary artery disease would be to reliably exclude significant stenosis and, thus, avoid unnecessary invasive conventional coronary angiography. To do this, a test should have, at clinically appropriate pretest likelihoods, minimal false-negative outcomes resulting in a high negative predictive value. However, little is known about the influence of patient characteristics on the clinical predictive values of coronary computed tomography angiography. Previous regular systematic reviews and meta-analyses had to rely on limited summary patient cohort data offered by primary studies. Performing an individual patient data meta-analysis will enable a much more detailed and powerful analysis and thus increase representativeness and generalizability of the results. The individual patient data meta-analysis is registered with the PROSPERO database (CoMe-CCT, CRD42012002780).

**Methods/Design:**

The analysis will include individual patient data from published and unpublished prospective diagnostic accuracy studies comparing coronary computed tomography angiography with conventional coronary angiography. These studies will be identified performing a systematic search in several electronic databases. Corresponding authors will be contacted and asked to provide obligatory and additional data. Risk factors, previous test results and symptoms of individual patients will be used to estimate the pretest likelihood of coronary artery disease. A bivariate random-effects model will be used to calculate pooled mean negative and positive predictive values as well as sensitivity and specificity. The primary outcome of interest will be positive and negative predictive values of coronary computed tomography angiography for the presence of coronary artery disease as a function of pretest likelihood of coronary artery disease, analyzed by meta-regression. As a secondary endpoint, factors that may influence the diagnostic performance and clinical value of computed tomography, such as heart rate and body mass index of patients, number of detector rows, and administration of beta blockade and nitroglycerin, will be investigated by integrating them as further covariates into the bivariate random-effects model.

**Discussion:**

This collaborative individual patient data meta-analysis should provide answers to the pivotal question of which patients benefit most from noninvasive coronary computed tomography angiography and thus help to adequately select the right patients for this test.

## Background

**Figure 1:**
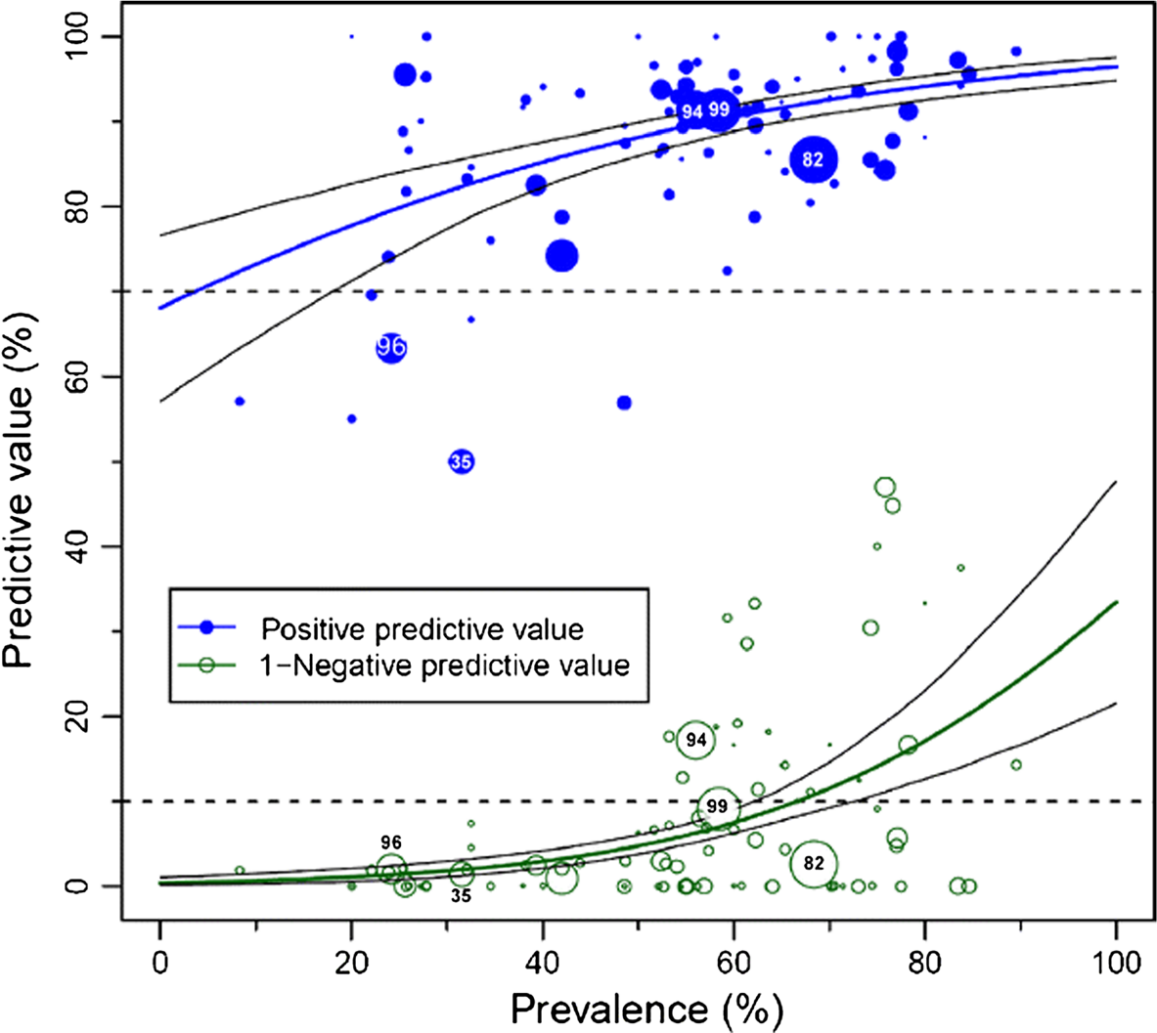
**Predictive values of coronary computed tomography angiography. **The population-averaged predictive values (based on published studies and their summary results) are shown as blue (positive) and green (negative) curves with their 95% confidence intervals based on the published studies (circles). The negative predictive values are shown as 1-negative predictive values to avoid overlap with the positive predictive value curve. The number of patients included in the published studies is indicated by the size of the circles. The dashed lines indicate the predefined minimal predictive values on the y-axis (90% for negative and 70% for the positive predictive values). Such analyses are limited because only the published predictive values in the studies and the calculated prevalences (but not the pretest likelihoods [7-9]) are available. Thus, individual patient data (IPD) from a collaborative meta-analysis can overcome these limitations and draw more meaningful conclusions about the clinical utility of diagnostic tests. Image reproduced from: Schlattmann P, Schuetz GM, Dewey M: Influence of coronary artery disease prevalence on predictive values of coronary CT angiography: a meta-regression analysis. Eur Radiol 2011, 21(9):1904–1913 [12].

Coronary artery disease (CAD) is the main cause of death in industrialized countries [1]. To allow early detection as well as accurately ruling out CAD, a reliable noninvasive test would have important advantages. To reliably exclude disease, a diagnostic test with a minimal proportion of false-negative results and thus a relatively high sensitivity should be used [2]. Coronary computed tomography (CT) angiography has already been evaluated comprehensively concerning its diagnostic accuracy potentials by single studies and meta-analyses. However, the influence of the clinical presentation, risk factors and prior test results of patients, which can be used to estimate the influence of the pretest likelihood of CAD on the positive and negative predictive values of coronary CT angiography, are not fully understood. A few single-center studies have been published dealing with the influence of the pretest likelihood of CAD on the diagnostic performance of CT. Meijboom *et al*. demonstrated in 254 symptomatic patients that the accuracy of coronary CT angiography was greatest in patients with low-to-intermediate pretest likelihood whereas the negative predictive value was substantially reduced in patients with high pretest likelihood [3]. This reduction of the negative predictive values in patients with high disease prevalence was also shown in a comparable study by Husmann *et al*. comprising 88 patients [4]. In 90 patients, Leber *et al*. also demonstrated that CT allows CAD to be accurately ruled out if an intermediate pretest likelihood is present [5]. Applying Bayesian analysis, Dewey *et al*. [6] confirmed that the diagnostic performance of both CT and magnetic resonance coronary angiography was influenced by the pretest likelihood. Pretest likelihood of CAD can be estimated according to clinical presentation and risk factors applying several tabulation tools [7-11] and the appropriateness of further testing may be estimated based on their results. Establishing and further elucidating the potential of pretest likelihood estimations to ensure the appropriateness of CT is an important aim of cardiovascular medicine. In a meta-regression analysis using disease prevalence as a surrogate marker for pretest likelihood, Schlattmann *et al*. further specified the boundaries of low-to-intermediate pretest likelihood for patients in whom coronary CT angiography can be applied as a reliable rule-out test [12] (Figure 1).

The analysis is based on summary patient collective data from primary study articles aggregated in a regular meta-analysis [13] and, thus, further specific patient-level information for more detailed subanalyses were not available.

The purpose and main aim of this project is thus to summarize the published and unpublished evidence on coronary CT angiography using IPD in a collaborative meta-analysis to draw more reliable and generalizable conclusions [14] concerning the appropriateness of CT in patients with varying presentations and thus pretest likelihoods [15]. The results should enable us to determine which patients benefit most from noninvasive coronary angiography and those less suited to undergo CT.

Moreover, the influence of additional patient and acquisition characteristics will also be summarized in our collaborative meta-analysis to allow us to draw important conclusions concerning the eligibility of patients for coronary CT angiography and the importance of specific technical requirements and methods of preparation. Among these factors being discussed or having previously been shown to influence the diagnostic performance of coronary CT angiography is the heart rate of the patients during scanning, which may lead to motion artifacts, and the body mass index, which, if severely increased, can result in rather noisy nondiagnostic test results [16-18]. Also, technical factors such as the number of simultaneous CT detector rows and the administration of beta blockade to reduce heart rate and nitroglycerin to dilate the vessels have been shown in single-center studies to alter the diagnostic performance and image quality of coronary CT angiography [19-24]. In detail, subanalyses will be performed to investigate the following questions:

· Is there a decrease in diagnostic accuracy of coronary CT angiography in patients with a body mass index >30?

· Does heart rate control (heart rate <60 beats per minute) lead to an increase of accuracy?

· Does the use of ≥64-row CTs lead to an increase of diagnostic accuracy?

· Is there a difference in diagnostic accuracy between men and women after adjusting for differences in pretest likelihood?

· Is diagnostic accuracy higher in patients >70 years of age or younger patients after adjusting for differences in calcium score?

· Is a certain Agatston calcium score threshhold a good universal predictor for coronary artery disease?

· Is diagnostic accuracy of CT different in patients with atypical or typical chest pain?

This IPD meta-analysis will enable us to perform the main and secondary analyses on an individual per-patient basis, the clinically most relevant level of analysis. The results may have relevant clinical implications because conventional coronary angiography (CCA) exposes the patients to infrequent yet considerable risks, such as myocardial infarction, coronary artery dissection and ventricular tachycardia [25,26], which might be avoided using CT in selected patient populations with suspected CAD. In addition, there are also economic considerations because CT might also be more cost effective [27].

## Methods/Design

This is a study protocol for the collaborative IPD meta-analysis on coronary CT angiography in comparison to conventional angiography as the gold standard, called Collaborative Meta-Analysis of Cardiac CT (CoMe-CCT). It was registered with the PROSPERO database [28,29] (registration number: CRD42012002780). This collaborative IPD meta-analysis is exempt from ethical approval as the analysis as well as the results for publication only involve de-identified data and all individual studies that will be summarized have received local ethics approval.

### Identifying eligible studies for CoMe-CCT

Valid published and unpublished studies will be retrieved by directly contacting all corresponding authors from published studies on coronary CT versus CCA in patients with suspected CAD. This will be achieved by including the primary studies of a recent regular systematic review and meta-analysis by our working group [13], and performing an update of this review to identify most recent studies. A sensitive search strategy using the particular thesaurus terms of the specific databases in combination with free text word synonyms [2] will be used to search MEDLINE (via PubMed), EMBASE (via Ovid) and ISI Web of Science databases for updating the article pool of relevant studies (suggested search strategies for all databases are provided in Additional file 1). We will also search bibliographies of retrieved studies, systematic reviews and meta-analyses, and include results of abstracts presented at most recent congresses as suggested by clinical experts.

Key inclusion criteria are a prospective study design; use of a contemporary CT scanner with at least 12 simultaneous detector rows; use of ≥50% diameter reduction as the cut-off criterion for significant coronary artery stenoses in CT and CCA; application of CCA in all patients, regardless of the results of CT; provision of individual patient results for CT and CCA (positive, negative, and nondiagnostic), which allow calculation of 2×2 (excluding nondiagnostic results) or 3×2 (including nondiagnostic results) tables [30]; articles published in English or German; and provision of basic patient characteristics (age, gender, angina type, heart rate).

Studies with the following characteristics will be excluded: retrospective study design; overlap with other studies as indicated by the corresponding author.

Study screening and selection will be performed by two independent investigators. Retrieved articles will be searched after duplicate papers (from MEDLINE, EMBASE and ISI Web of Science) are identified and eliminated. In a first phase, titles and abstracts will be scanned and, in accordance with the inclusion and exclusion criteria, either discarded or retained for further investigation. In a second phase, the remaining potentially eligible articles will be reassessed in depth using the full text.

### Methodological quality assessment

Two investigators will independently carry out the data extraction (including quality assessment) from all the retrieved published studies based on the full text articles. Discrepancies will be resolved in a consensus meeting, or, if agreement cannot be reached, they will be resolved in consensus with a third investigator. To assess and quantify the inter-rater agreement between the two investigators in evaluating methodological quality using the QUADAS tool [31], we will calculate the kappa statistics. The data extracted will be: general and detailed methodological study characteristics, characteristics of the study population, details of the CT technology used, and detailed reference standard (CCA) specifications.

### Collection of individual patient data

An international steering committee of renowned experts from the field of noninvasive coronary CT angiography has been established to lead, oversee and represent the CoMe-CCT project. The steering committee consists of four clinical experts for cardiac CT: Stephan Achenbach, Erlangen, Germany; Matthew Budoff, Los Angeles, CA, USA; Mario J Garcia, New York, NY, USA; Marc Dewey, Berlin, Germany, and one clinical expert for CCA: Michael Laule, Berlin, Germany. The steering committee is completed by the project’s statistician Peter Schlattmann, Jena, Germany.

Corresponding authors of identified eligible published studies will be contacted using mail including a cover letter detailing the objectives of the collaborative meta-analysis, background information on IPD meta-analysis, and a CD containing a data collection file for input of individual patient results for the project. The details of the obligatory and additional patient and study characteristics are shown in Tables 1 and 2. The filled out data templates will be send back to us by either mail on CD or, more conveniently, by email. Further communication will mainly be performed by email or phone. Corresponding authors will also be contacted about unpublished data that will be eligible for inclusion in the collaborative meta-analysis if the inclusion criteria are met. To further enlarge the clinical data pool, authors will additionally be asked to provide data from registries. These data will not be included in the primary analyses, which are only based on data from the prospective studies.

**Table 1:** Obligatory computed tomography technology and patient characteristics requested in the data file

**Computed tomography characteristics**	**Patient and conventional coronary angiography characteristics**
Publication from which the data is derived	Age and gender, type of symptoms (typical angina pectoris, atypical angina pectoris, chest pain, no pain, pain unknown)
Results of CT (positive^a^, negative, nondiagnostic^b^). Comment and explanation for nondiagnostic patients	Results of conventional coronary angiography (positive^a^, negative, nondiagnostic^b^). Comment and explanation for nondiagnostic patients

^a^At least one ≥50% stenosis in one vessel; ^b^no stenosis in any vessel and at least one vessel not fully evaluable.

**Table 2:** Additional computed tomography technology and patient characteristics requested in the data file

**Additional computed tomography characteristics**	**Patient characteristics and additional tests**
Effective radiation dose	Weight and height
Type of electrocardiographic gating	Derived body mass index
Number of detector rows used	Calcium score, heart rate during scanning, presence of cardiac risk factors (hypertension, diabetes, hyperlipidemia, current or former smoker, positive family history, prior myocardial infarction)
Beta blockade (type, route, dosage)	Results of rest electrocardiography
Nitroglycerin (type, dosage)	Results of stress electrocardiography
Contrast agent (type, concentration, flow, amount)	Results of stress echocardiography
Breath hold duration	Results of stress scintigraphy

### Data management, security and validation

The same two investigators who will perform the electronic database search and identify published eligible studies for the regular review will also collect and assemble the IPD provided by the investigators of published and unpublished studies. Data will be sent as email attachments to the coordinating investigator of CoMe-CCT in an anonymized and de-identified way (without directly identifying information, that is, record numbers, names, addresses and so on will be removed). Data will be accepted in any kind of electronic format (for example, SPSS, Stata, Word document,) but, ideally, the investigators will provide the data using the individualized Microsoft Excel (Microsoft Corp., Redmond, WA, USA) spreadsheets made available to them. The original data collection files sent by the authors will be kept in their original version and will be saved on a password-protected server behind the firewall in order to ensure security. Only the coordinating laboratory of CoMe-CCT at Charité will have direct access to the individual data. Both investigators will each perform data validation using a copy. To ensure quality of the data, they will independently check the received data sets for data entry mistakes and consistency and will compare the sum of the individual patient results received with the published summary results from the primary studies. Differences will be discussed and settled in consensus, if possible. They will contact the providing corresponding author in case of discrepancies that should be resolved.

### Outcome measures and statistical analysis

An extension of the exact binomial rendition [32] of the bivariate mixed-effects regression model developed by van Houwelingen *et al*. [33,34] for treatment trial meta-analysis and modified for synthesis of diagnostic test data [35,36] will be used for data synthesis. This bivariate statistical model does not transform pairs of positive and negative predictive values into a single indicator, but preserves the two-dimensional nature of the data taking into account any correlation between the two. Given that we are dealing with individual data, a three-level bivariate random-effects model will be applied. On the first level of the model, a binomial distribution will be assumed for the true positives among the test positives and the true negatives among the test negatives. On the second level, the variability between patients within a certain study will be modeled. Finally, on the third level the variability between studies will be taken into account.

The primary covariate of interest is the pretest likelihood of CAD. It will be calculated using a validated prediction tool [10] based on the original Diamond and Forrester model for risk calculation [7]. Using this tool, the pretest likelihood of a patient based on age, gender and clinical presentation can be estimated. Furthermore, the influence of covariates such as heart rate and body mass index of patients as well as the number of CT rows, and the administration of beta blockade and nitroglycerin, will be taken into account on the individual patient level. Model selection will be based on the likelihood ratio test and Bayesian Information Criterion.

Based on this model, mean logit positive predictive values and negative predictive values with their standard error and 95% confidence intervals, estimates of the between-study variability in logit positive and negative predictive values, and the covariance between them will be estimated. These quantities will be back-transformed to the original scale to obtain summary positive and negative predictive values. Using this data, a statistical prediction for a new cohort will be performed following the ideas presented by Skrondal and Rabe-Hasketh [37,38]. Estimation will be done with SAS 9.2, Proc GLIMMIX and STATA 11, using the package GLLAMM. In addition, for performing a sensitivity analysis, we will also use WinBUGS [39]. If necessary, macros will be written to facilitate the use of three-level generalized linear mixed-effects models.

Based on the model described above, mean logit sensitivity and specificity with their standard error and 95% confidence intervals, estimates of the between-study variability in logit sensitivity and specificity, and the covariance between them will be estimated. These quantities will be back-transformed to the original ROC (Receiver Operating Characteristic) scale to obtain summary sensitivity, specificity and the diagnostic odds ratios. The derived logit estimates of sensitivity, specificity and respective variances are then used to construct a hierarchical summary ROC curve for CT with summary operating points for sensitivity and specificity on the curves and a 95% confidence contour ellipsoid.

Here the individual patient-specific covariates such as type and severity of symptoms, body mass index, beta blockade and sublingual nitroglycerin will be used in the regression analysis. This serves as tool to identify spectrum bias and clinically relevant influential factors.

For performing sensitivity analysis, the analysis will be redone by leaving out one study. The generalized linear mixed model is known to be sensitive to starting values. Thus an additional analysis with Bayesian methods using PROC MCMC, SAS 9.2 will be performed.

## Discussion

We believe that this collaborative multi-centric and multi-continental IPD meta-analysis will have an important impact on the clinical management of patients with suspected CAD as it should provide an answer to the question of which patients benefit most from coronary CT angiography. Furthermore, additional factors that influence the diagnostic performance of CT in comparison to CCA can be identified in this meta-analysis. This IPD meta-analysis also holds the potential to analyze and compare the predictive value of cardiac CT and CCA for subsequent major adverse cardiovascular events. Finally, the CoMe-CCT may facilitate the adequate selection of patients for cardiac CT by estimating the pretest likelihood for disease and predicting the diagnostic performance for individual patients that should ultimately help to avoid unnecessary examinations and thus decrease the use of scarce health care resources in the future [40].

## Trial status

Currently, in the course of performance of the IPD meta-analysis, we are in the state of data collection and data validation. No statistical analysis has yet been performed. The update search still has to be performed to be able to identify most recent articles and to contact the corresponding authors. At the time of study protocol submission, 54 single data sets from 40 authors and including more than 5,500 IPD comparing CT and CCA have been acquired.

## Competing interests

PS and MD are supported by a grant of the German Federal Ministry of Education and Research (BMBF) for meta-analyses as part of the joint program "clinical trials" of the BMBF and the German Research Foundation (DFG). PS is also supported by another grant of the DFG (Schl 3–1) and has received lecture fees from Bayer-Schering. MD has received grant support from Heisenberg Program of the DFG for a professorship (DE 1361/14-1), European Regional Development Fund (20072013 2/05, 20072013 2/48), German Heart Foundation/German Foundation of Heart Research (F/23/08, F/27/10), Joint program from the DFG and the BMBF for meta-analyses (01KG1013, 01KG1110), GE Healthcare, Bracco, Guerbet, and Toshiba Medical Systems and lecture fees from Toshiba Medical Systems, Guerbet, Cardiac MR Academy Berlin, and Bayer (Schering-Berlex). MD is a consultant to Guerbet and one of the principal investigators of multi-center studies (CORE-64 and 320) on coronary CT angiography sponsored by Toshiba Medical Systems. He is also the editor of *Coronary CT Angiography* and *Cardiac CT*, both published by Springer, and offers hands-on workshops on cardiovascular imaging (http://www.ct-kurs.de). Institutional master research agreements exist between Charité and Siemens Medical Solutions, Philips Medical Systems, and Toshiba Medical Systems. GMS is a physician working as a research assistant in MD's working group. His salary is financed by a grant from the BMBF for meta-analyses granted to MD. RR is a research assistant in MD's working group; his salary is also paid by a grant from the BMBF for meta-analyses granted to MD. SA is Speaker Honoraria and receives grant support from Siemens AG; he is a consultant for Servier and Guerbet. MB receives grant support from GE. GP has received a grant from GE Healthcare as speaker's bureau. JH received research grants from Siemens Medical Solutions. UJS is a consultant for and receives research support from Bayer, Bracco, GE, Medrad, and Siemens. AD and HM have received grants from Region Syddanmark (grant number 08–17862 and 09–13526). DAH reports institutional research and development agreements with Philips Medical Systems. KAØ has received grant support from Vejle Hospital Research Foundation and a PhD scholarship from the Faculty of Health Sciences at Aarhus University. GRU has received lecture fees from Siemens Medical Solutions and Philips Medical Systems, but has no other conflicts of interest. KN is supported by an Erasmus MC Fellowship, receives unrestricted research support from Siemens Medical Solution and Bayer Scheering, and lecture fees from Siemens Medical Solutions, Bayer Scheering and Abbott Vascular. KN receives royalties for lectures including service on speakers' bureaus from Bayer Healthcare and Siemens Healthcare. TRCJ is supported by research grants and receives lecture fees from Siemens Healthcare and Bayer Healthcare. BJWC receives research support from GE Healthcare and educational support from TeraRecon. MJG, WBM, DA, HA, LH, SL, BLG, AAS, AS, EZ, VMR, AH, BLN, HB, SMMJ, BAH, JR, MS, CL, EM, AR, AJHAS, RPM, JCT, SM, DM and ML have no competing interests to declare. NB certifies that there is no conflict of interest with any financial organization regarding the material discussed in the manuscript. MFLM declares that there are no conflicts of interest for himself (or for any other Utrecht authors (Prokop, Cramer, Doevendans and De Vos) in the study described in Meijboom, Meijs, JACC 2008).

## Authors’ contributions

MD conceived the idea; MD, GMS, RR and PS drafted the initial protocol. All authors reviewed, commented on, read and approved the final manuscript.

## Authors’ information

GMS is a physician working as a junior research assistant in MD's working group. His research interests and publications include the conduction of systematic reviews and meta-analyses as well as quality aspects concerning reporting and methodology of diagnostic accuracy studies in radiology.

PS is a physician and a statistician and professor of medical statistics. His research interests are modeling of heterogeneity in medical research including meta-analytic methods. PS is author of the book *Medical Applications of Finite Mixture Models*.

SA (MD) is the chairman of the Department of Cardiology. His main are of research is cardiac imaging, with a special focus on CT and noninvasive assessment of coronary atherosclerosis. He has authored approximately 150 original research papers and is associate editor of *JACC Cardiovascular Imaging* and the *Journal of Cardiovascular CT*. He has served as the chairman of the Society of Cardiovascular CT, and is currently a board member of the Society of Cardiovascular CT as well as the European Society of Cardiology.

MB (MD) is a cardiologist who has over 20 years’ experience with early detection of coronary artery disease using both coronary artery calcium testing and CT angiography. He has published four books on the topic of cardiac CT and published over 450 MEDLINE-indexed articles on cardiovascular CT. He is past president of both the Society of Atherosclerosis Imaging and Prevention and Society of Cardiovascular Computed Tomography. He is currently a professor of medicine at the David Geffen School of Medicine at UCLA, and program director of Cardiology at Harbor-UCLA Medical Center.

MJG (MD) is chief of Division of Cardiology at the Montefiore Medical Center and professor of medicine at the Albert Einstein College of Medicine. He is member and past founding board member of the Society of Cardiovascular CT and member of the American Board of Cardiovascular Disease. His research interests include screening for CAD, management of chest pain in the emergency department, and imaging in heart failure.

RR holds a Master in Education and is currently enrolled in the Master of Science in Statistics Program. He works as an assistant researcher in the working group of MD and his research interests include biometrics as well as survey statistics.

GP graduated in medicine followed by postgraduate degree in cardiology and radiology at University of Milan. From 1993 to 1994 he worked as visiting researcher at the Institute of Human Physiology focusing his basic research in the field of cardiovascular diseases. From 1995 to 2000 he began scientific and clinical training in noninvasive cardiology. During this time, he has devoted much of his activity in clinical research in the field of dilated cardiomyopathy and in 2000 he published his first scientific book. In 2001 he began training in cardiovascular imaging and in 2004 he participated in the founding of one of the first cardiac radiology services in Italy. He is currently director of Magnetic Resonance Imaging (MRI) and deputy director of the CT Unit. He has authored more than 65 MEDLINE-indexed articles in international journals, four scientific books and more than 150 scientific abstracts at national and international meetings in the field of dilated cardiomyopathy and of cardiovascular imaging. He is also reviewer of *European Radiology*, *International Journal of Cardiology*, *Journal of Cardiovascular Medicine*, *Expert Review of Cardiovascular Therapy*, *Case Reports in Cardiology*, *Case Reports in Medicine*, *International Journal of Cardiovascular Imaging* and *European Heart Journal - Cardiovascular Imaging*.

WBM (MD, PhD) is a resident cardiologist and researcher. His research interests and publications include diagnostic performance of CT coronary angiography.

DA is a physician with degree in cardiology and radiology and director of the Cardiovascular CT Unit. His research interests and publications mainly lie in the different applications of CT coronary angiography, such as patients with coronary stents or by-pass grafts, with dilated cardiomyopathy of unknown etiology, with coronary anomalies and with diabetes.

HA (MD, MPH, EBCR) is an associate professor and senior radiologist. He is a board certified radiologist and board certified neuroradiologist. His main research interests are body CT, emergency radiology and abdominal imaging.

LH (MD) is a cardiac CT research fellow. Her research interests include non-calcified plaque, plaque volume analysis and the effect of hormone replacement therapy in perimenopausal women.

NB is a cardiologist consultant, responsible for the Cardiovascular CT and Cardiac MR Program, working in collaboration with the Cardiovascular Research and Development Unit of the Faculty of Medicine of Porto. His research interests and publications are related to noninvasive detection of CAD and risk stratification.

JH (MD) is a board certified doctor for internal medicine and cardiology. He is currently vice director of the Medizinische Klinik I. He is a professor of medicine at the Ludwig-Maximilians-Universität München. As an interventional cardiologist, his clinical main focus includes the percutaneous treatment of coronary and valvular diseases. In addition, he has gained an international reputation as a noninvasive cardiologist focusing on noninvasive coronary imaging by CT imaging. He is an active member in several societies for cardiology and cardiovascular CT imaging (joerg@hausleiter.com).

SL is a senior staff radiologist working as the section head of CT and Emergency Radiology. His research interests are cardiovascular imaging, abdominal radiology, emergency radiology and forensic imaging. SL is editor of two books and author of more than 130 original and review articles and more than 20 book chapters.

BLG (MD, PhD) is a cardiologist and researcher working in the field of noninvasive cardiac imaging. BLG has published over 70 MEDLINE-indexed articles including works on cardiovascular imaging, cardiac MRI, CT and nuclear imaging. He is the treasurer of the working group for cardiovascular MRI of the European Heart Association.

MFLM works as a resident. His clinical and research activities focus on cardiac CT and MRI, left ventricular hypertrophy and genetics.

AAS (MD) is a radiologist and associate professor of radiology. He is currently chairman of the Radiology and Cardiac CT Departments and his publications and particular interests are mainly related to cardiothoracic and body imaging. He has 14 MEDLINE-indexed and 18 ISI Web of Knowledge-indexed published papers, mostly in the field of cardiothoracic and vascular imaging. He is head of the Cardiac Imaging Committee in the Iranian Society of Radiology and elected head of the Society of Cardiovascular CT in Iran and a member of the Iranian Board of Radiology. He is the head of the Cardiac Imaging Section in Advanced Diagnostic and Interventional Radiology center and is an editorial board member as well as associate editor of the Iranian Journal of Radiology (abarjshabestari@yahoo.com).

AS is an interventional cardiologist and researcher in the field of cardiovascular imaging and an associate professor. His research interests are in the field of cardiac remodeling after acute myocardial infarction and coronary plaque morphology on cardiovascular imaging.

EZ (MD) is a radiologist in MD's working group and her research interests include all aspects of cardiovascular imaging.

UJS is a professor of radiology, cardiology and pediatrics and the director of Cardiovascular Imaging. His main scientific interest is cardiovascular imaging, especially the use of advanced CT and MRI techniques for diagnosing heart disease. UJS serves on the editorial boards of several scientific journals including *Radiology*, the J*ournal of the American Heart Association*, the *American Journal of Roentgenology*, *Academic Radiology* and *European Radiology* and is Associate Editor of the *Journal of Thoracic Imaging*. He has authored >230 articles in peer-reviewed scientific journals, 20 book chapters, and three books. UJS is a member of numerous scientific societies and serves as chairman of several committees of the American College of Radiology, the Radiological Society of North America, American Heart Association, North American Society of Cardiovascular Imaging, Society of Computed Body Tomography and MR, and the Society of Thoracic Radiology. He is an honorary member of the Société Canadienne-Française de Radiologie as well as of the Hungarian Radiological Society and was elected Fellow of the American Heart Association, of the Society of Computed Body Tomography and MR, and of the Society for Cardiovascular CT.

AD is a consultant and associated professor. His main interest is in the area of atherosclerosis with focus on prevention of ischemic heart disease. Since 2006, he has been working with cardiac CT, and is now the chairman of the Danish working group Cardiac Imaging. AD is the principal investigator of the multi-center study, DanRisk, which aims to examine the prevalence of coronary calcification in middle-aged people and to compare the CAC score with the HeartScore.

DAH is a clinical cardiologist and director of Interventional Cardiology. His clinical and research interests include predictive value of plaque level analysis of coronary CT angiograms, the clinical role of cardiac CT angiography, interventional and therapeutic studies in acute coronary syndromes and transcatheter aortic valve implantation.

VMR (MD, PhD) is a cardiology physician, assistant professor and researcher. He is chief of the CT Department and full member of the Cuban Society of Cardiology. His research interests and publications are related to noninvasive and invasive methods of cardiovascular diseases diagnostic. He has more than 40 national and international publications on these subjects (Vladimir@icccv.sld.cu).

AH is a senior cardiologist and researcher with experience in the field of cardiac CT and MRI. Between 2006 and 2009, he held a fellowship in the field of cardiac CT and MRI at the German Heart Institute Berlin.

BLN is a cardiologist and researcher with longstanding experience in the field of cardiac diagnostic imaging. He is currently working as an associate professor and senior consultant. He has published more than 50 MEDLINE-indexed articles including works on cardiovascular CT imaging.

HB is a radiologist. His research interests include multiple aspects of cardiac CT, most notably noninvasive coronary angiography, assessment of myocardial viability and plaque analysis.

KAØ is a fellow in Cardiology at Vejle Hospital and Odense University Hospital, departments of Cardiology, Vejle and Odense, Denmark. His research and PhD have focused on the use of coronary CT angiography in patients with suspected CAD.

SMMJ (MD, MRCP, BSc(Hons)) gained her degrees in Science and Medicine from the University of Glasgow in 2000 and 2002 respectively. She subsequently underwent clinical training in medicine and cardiology and, following research in the field of cardiac imaging, was awarded a postgraduate Doctor of Medicine degree in 2011, again conferred by the University of Glasgow. She has worked as a specialist registrar in clinical cardiology since 2008 and her subspecialty interests, research and publications are focused on cardiac imaging and heart failure.

BAH is a clinical staff cardiologist. His primary research interests are in cardiac imaging, cardiac CT and cardiac MRI.

JR is a physician working as a clinical and research fellow. His key activities both in clinical routine and science are cardiac CT as well as cardiac MRI. During the enrolment period of CoMe-CCT, JR was in charge of patient inclusion and patient data acquisition at Kerckhoff Heart and Thorax Center in Bad Nauheim, Germany.

MS is professor of radiology. His clinical and research interest is cross-sectional imaging.

CL is a cardiologist; he is American College of Cardiology Level 3 certified for cardiac CT and a Fellow of the Society of Cardiovascular Computed Tomography.

EM (MD, FESC) is associated professor of cardiology. He is chief of the catheterization laboratory (UOS of hemodynamics). His principal field of interest is interventional cardiology and imaging by CT. EM is author of 62 MEDLINE-indexed articles including works on coronary revascularization (CABRI and SOS trials), HCMO, radiation (PROTECTION trial), studies comparing CCA and CT. He is author of two chapters of the book *Cardiac CT*. He is chairman of the working group of cardiac CT and nuclear cardiology of the Italian Society of Cardiology.

AR is a radiologist and researcher in the field of diagnostic imaging and particularly in cardiac imaging. He is chief of the Simple Operative Unit in Cardiac Radiology. AR has published over 30 MEDLINE-indexed articles including works on cardiovascular imaging, interventional radiology, radiation and abdominal radiology. He is author of several book chapters concerning cardiac radiology.

AJHAS (MD, PhD) is a cardiologist. His clinical and research interests include noninvasive cardiac imaging, diabetes mellitus and aortic diseases.

RPM is a medical student, working as a research assistant under the supervision of Konstantin Nikolaou, MD and Fabian Bamberg, MD, MPH. His research interests include cardiovascular CT and cardiovascular MRI.

GRU is a radiologist with main occupation at the private Aleris Hospital in Oslo, working mainly with breast diagnostics. He is now a part-time PhD student at Akershus University Hospital, researching cardiac imaging.

KN is a cardiologist whose clinical and research interests include noninvasive cardiac imaging and acute cardiac care.

HM is consultant, professor and head of research. His main interest is in the area of ischemic heart disease. HM is the creator of Odense Chest Pain Bio-bank, which was established in 2010 to 2011 and includes 2,500 patients with a suspected acute myocardial infarction.

KN is a full professor of radiology, vice chair of the Department of Clinical Radiology, and section chief of MRI. His research interests include cardiovascular CT/dual energy CT, cardiovascular MRI, imaging of atherosclerosis, thoracic imaging and molecular imaging. KN has published more than 160 scientific papers, one book, and more than 20 book chapters. He also serves as a reviewer for numerous journals, including the *American Journal of Radiology*, *Circulation*, *JACC Imaging*, *European Journal of Radiology*, *Investigative Radiology*, *International Journal of Cardiovascular Imaging*, *Journal of Computer Assisted Tomography*, *Journal of the American College of Cardiology*, *Magnetic Resonance in Medicine*, and others.

JCF (MD) is a cardiologist and director of the research center of the Montreal Heart Institute. He also leads the Canadian Atherosclerosis Imaging Network and holds the Université de Montréal-endowed research chair in atherosclerosis and Canada Research Chair in translational and personalized medicine.

TRCJ is a radiologist working as associate professor and head of CT. Among his research interests are cardiovascular CT and MRI and spectral CT. He has published some 75 peer-reviewed articles and three radiological textbooks.

SM is an interventional cardiologist working in the CathLab of the Department of Cardiology. His research interests include interventional cardiology and cardiovascular imaging (simonemuraglia@yahoo.it).

BJWC is associate professor and staff cardiologist. He is the co-director of Cardiac Radiology and the director of the Postgraduate Cardiac Imaging Program. His research focuses on developing and evaluating novel imaging techniques with the goal of understanding their clinical applications and benefit to patient care.

DM is chairman of the Department of Radiology. His main research interests are cardiovascular imaging and image guided interventions.

ML is a senior physician and deputy director of the cardiac catheterization laboratory at the Department of Cardiology

MD is a radiologist and researcher with vast experience in the field of diagnostic imaging and chief consultant . MD has published over 120 MEDLINE-indexed articles including works on cardiovascular imaging, claustrophobia, cost-effectiveness, meta-analyses, radiation and patient safety. He is an associate editor of *Radiology* and *European Radiology* and the editor of the books *Coronary CT Angiography* and *Cardiac CT*. He is also president of the 1898-founded Röntgen Society of Berlin and Brandenburg.

PS, SA, MB, MJG, ML and MD are members of the steering committee of CoMe-CCT.

## Supplementary Material

Additional file 1 Search Strategies for MEDLINE, EMBASE and ISI Web of Science. Click here for file
