# Supplementary material for: Individual patient data meta-analysis for the clinical assessment of coronary computed tomography angiography: protocol of the Collaborative Meta-Analysis of Cardiac CT (CoMe-CCT)
Source: Syst Rev. 2013 Feb 15;2:13. doi: 10.1186/2046-4053-2-13 (PMC3576350; doi:10.1186/2046-4053-2-13)
Supplement: Additional file 1 — Search Strategies for MEDLINE, EMBASE and ISI Web of Science. [file 2046-4053-2-13-S1.doc]

a) MEDLINE (via PubMed)

| **Search areas** | **Terms** |
| --- | --- |
| **Index test** | (("tomography, x-ray computed"[MeSH Terms]) |
|  | OR ("computed tomography"[Text Words]) |
|  | OR ("CT"[Text Words]) |
|  | OR ("multidetector"[Text Words]) |
|  | OR ("multi-detector"[Text Words]) |
|  | OR ("multi detector"[Text Words]) |
|  | OR ("MDCT"[Text Words]) |
|  | OR ("multislice"[Text Words]) |
|  | OR ("multi-slice"[Text Words]) |
|  | OR ("MSCT"[Text Words]) |
|  | OR ("dual-source"[Text Words]) |
|  | OR ("dual source"[Text Words]) |
|  | OR ("DSCT"[Text Words]) |
|  | OR ("multi-row"[Text Words]) |
|  | OR ("multi row"[Text Words])) |
|  | **AND** |
| **Procedure** | (("coronary angiography"[Mesh Terms]) |
|  | OR ("coronary angiography"[Text Words])) |
|  | **AND** |
| **Condition under investigation** | (("coronary disease"[Mesh Terms]) |
|  | OR ("coronary artery disease"[Mesh Terms]) |
|  | OR ("coronary stenosis"[Mesh Terms]) |
|  | OR ("coronary disease"[Text Words]) |
|  | OR ("coronary artery disease"[Text Words]) |
|  | OR ("coronary stenosis"[Text Words]) |
|  | OR ("CAD"[Text Words]) |
|  | OR ("coronary heart disease"[Text Words]) |
|  | OR ("CHD"[Text Words])) |

**b) EMBASE (via Ovid)**

| exp computed tomographic angiography/ |
| --- |
| OR computed tomograpic angiography.tw. |
| OR computed tomography coronary angiography.tw. |
| OR computed tomographic coronary angiogaphy.tw. |
| OR coronary CT angiography.tw. |
| OR CTA.tw. |
| OR CTCA.tw. |
| OR CCTA.tw. |
| **AND** |
| (exp coronary artery disease/ |
| OR exp coronary artery obstruction/ |
| OR ("coronary disease".tw.) |
| OR ("coronary artery disease".tw.) |
| OR ("coronary stenosis".tw.) |
| OR ("CAD".tw.) |
| OR ("coronary heart disease".tw.) |
| OR ("CHD".tw.)) |

**c) ISI Web of Knowledge (Web of Science)**

| TS=("computed tomography" |
| --- |
| OR CT |
| OR "computer assisted tomography" |
| OR multidetector |
| OR multi-detector |
| OR "multi detector" |
| OR MDCT |
| OR multislice |
| OR multi-slice |
| OR MSCT |
| OR dual-source |
| OR "dual source" |
| OR DSCT |
| OR multi-row |
| OR "multi row") |
| **AND** |
| TS=("coronary angiography") |
| **AND** |
| TS=("coronary disease" |
| OR "coronary artery disease" |
| OR "coronary stenosis" |
| OR CAD |
| OR "coronary heart disease" |
| OR CHD) |
